# Treatment by untrained providers among sick infants in rural Odisha, India

**DOI:** 10.1017/S1463423619000380

**Published:** 2019-06-26

**Authors:** Mousumi Samal, Nabin Khara, Sanghamitra Pati, Krushna Chandra Sahoo

**Affiliations:** 1 Regional Medical Research Centre, Indian Council of Medical Research, Bhubaneswar, India; 2 District Rural Development Agency, Koraput, Odisha, India

**Keywords:** diagnosis, non-formal providers, referral services, treatment

## Abstract

**Aim:**

This study assessed the diagnosis, treatment and referral service provided by untrained providers for sick infants.

**Background:**

In rural India, lack of trained providers causes inopportune treatment of sick infants and results in increase in child morbidity and mortality. The untrained providers deliver a significant proportion of health care for rural infants; however, there is a paucity of information on their treatment practice.

**Method:**

A cross-sectional study was conducted in three rural blocks of Odisha. A total of 337 prescriptions recommended for sick infants were collected from the 15 untrained providers using pre-designed prescription form – designed as per the Integrated Management of Neonatal and Childhood Illness (IMNCI) guideline. The forms were collected through the periodic visit and regular follow-up to the providers.

**Findings:**

A total of 68% of infants were diagnosed with the possible serious bacterial infection, 56% fever, 10% feeding problems, 9% dysentery and 9% local bacterial infection. A total of 61% of sick infants prescribed antibiotics – cephalosporin was commonly prescribed (56%). Among severe persistent diarrhea-diagnosed infants, 76% prescribed oral rehydration salt (ORS), 48% zinc and 62% of them received various antibiotics. The untrained providers referred 23% of sick infants to trained providers/facilities. In rural settings, most of the sick infants sought care from untrained providers; however, none of them followed any standard treatment protocol. This study suggests there is a need for training on common disease algorithm and treatment using a standard guideline for untrained providers to reduce inopportuneness in the treatment of sick infants, promoting early diagnosis and referral services to public health systems.

## Background

The Sustainable Development Goals (SDG3) focused on comprehensive primary health care (Pettigrew *et al*., 2015). The Alma Ata Declaration promotes comprehensive primary health care addressing social determinants of health through inter-sectoral integration, community participation, implementation and regulation (Topp and Abimbola, 2018). ‘People at the center of healthcare’ is one of the key principles outlined in Alma Ata Declaration. Community participation and community workers revitalize to strengthened primary care and first-referral services (Walley *et al*., 2008).

The key characteristics of access include acceptability, approachability, availability, affordability and appropriateness (Corscadden *et al*., 2017; Jacobs *et al*., 2011). Access to primary health care is allied with cost-effectiveness and equity of health systems considering the underprivileged populations (Corscadden *et al*., 2017). While access to primary care is often demarcated with timeliness, distance and costs, it can be considered broadly as the capacity of the general public for obtaining appropriate services as per their need (Jacobs *et al*., 2011; Corscadden *et al*., 2017). Roadblocks for accessing primary care can happen either due to the services gap or due to the choice of people to seek care. In the absence of required number of trained providers in rural setting (Sharma, 2015), a parallel health-care system has been run by untrained providers (Datta, 2013).

In India, the private sector accounts for two-thirds of human resources and health facilities, and among them, more than half are untrained providers (Gautham *et al*., 2014). Like other low- and middle-income countries, in India, the untrained providers deliver a significant proportion of health care to rural, poor and vulnerable populations (Bloom *et al*., 2011). The untrained providers are often called informal health-care providers, non-formal providers, unlicensed providers, traditional healers, village doctors and ‘quacks’ (Bloom *et al*., 2011; Sudhinaraset *et al*., 2013). They are not registered with any government regulatory body and operate outside of the purview of regulation (Bloom *et al*., 2011). The untrained providers were working long hours, provide doorstep services to the community and often accessible on foot in affordable cost (Sudhinaraset *et al*., 2013; May *et al.*, 2014). In Odisha, more than 70% of the population live in rural areas. Like elsewhere in India, due to the lack of trained providers at primary health centers, most of the people seek primary care from private untrained providers (Dror *et al*., 2011). The unavailability of qualified health-care providers created space and opportunity for untrained providers, and they provide treatment to the rural people including children (Dror *et al*., 2011; George & Iyer, 2013); however, they often provided inappropriate treatment and were delaying the referral service (Sudhinaraset *et al*., 2013).

Appropriate and timely treatment and referral service are significant to improve child health services in rural and remote areas. In rural India, lack of trained prescribers often results in inopportune treatment, which upsurges the infant morbidity and mortality rates (Bloom et al., 2011; Dror *et al*., 2011; Pathak *et al*., 2011). The untrained providers are delivering a significant proportion of primary health care to rural infants. However, there is little known about their treatment protocol and practice for sick infants. Therefore, we assessed the diagnosis, treatment and referral service provided by untrained providers for sick infants.

## Methods

This cross-sectional study was conducted in three administrative blocks of Odisha state – Lamtaput and Nandapur blocks of Koraput, and Balianta block of Khurdha. These blocks were selected based on the feasibility, geographical location and urbanization.

As per census 2011, Lamtaput block consists of a total of 188 villages and the population is 59 873; among them 17% are children under five years (*n* = 10 363), and the literacy rate is 35%. Nandapur block consists of a total of 204 villages and the population is 72 579; among them 17% are children under five years (*n* = 12 215), and the literacy rate is 40%. Both these blocks of Koraput have around 50% population of scheduled tribe and above 14% of scheduled caste, and 100% population live in rural areas and most of them dependent on agriculture and collection of forest product for their livelihood. Balianta block consists of a total of 80 villages and the population is 91 728; among them 11% are children under five years (*n* = 9695), and the literacy rate is 84%, and has 28% scheduled caste and around 2% scheduled tribe. Out of the total population, 86% live in rural areas and mostly dependent on agriculture (Census, 2011).

The participants in this study were untrained providers, those who were providing allopathic medicine without any specific medical training. In the context of the Indian health system, the untrained doctors are neither a part of the public health system nor chosen by the system. However, due to a shortage of trained providers, a parallel health care system has been run by non-degree allopathic providers especially in rural and remote areas; they are providing more than 70% of the health care in rural India (Datta, 2013). They are largely accepted by the community because of their accessibility and affordability; they chose this profession as an income source. The providers were recognized by informal talking with the local leaders, pharmacy stores and also from the identified untrained providers. The recruitment criteria for this study was untrained providers those who do not have any formal medical or paramedical training. From the above criteria and process, a total of 15 providers participated in the study – 11 from Koraput and 4 from Khurdha.

For sample size calculation, we considered referral services of untrained providers as the primary outcome. Sample size was calculated based on a previous study (Kanjilal, 2013), which shows that a referral rate of a sick infant by untrained providers is 10%. By increasing the prevalence of reference to 30%, population 7000 as per census 2011, 95% confidence interval and design effect as one in open epi tool, the required sample size was 309 prescriptions. A total of 337 prescriptions were collected from two districts – 270 from Koraput and 67 from Khurdha.

The information of the providers was taken after they were agreed to participate in the study. It contained the education, occupation, daily and weekly patient load, daily and weekly sick infant visiting them, distance from sub-center, primary health center, community health center, sub-divisional and district headquarter hospital or capital hospital. The data collection form which was provided to untrained providers contained basic information about the sick infants and some basic measurements like height, weight and temperature. Commonly occurring signs and symptoms among sick infants were listed according to the Integrated Management of Neonatal and Childhood Illness (IMNCI) guideline (Govt. of India, 2005). Duration of illness and the previous treatment if any were mentioned in the form. There was no option list for treatment and it was left blank to be filled by the providers according to their treatment pattern. There was an option for referral services, that is whether there is any referral or not, and if yes, then where. One sheet was used for one infant and if repetition, the providers has to specify the number of visits. The commonly occurring signs and symptoms are listed according to IMNCI guideline.

The provider characteristics were collected after taking the consent of the untrained providers at once and the prescription books were provided to the providers considering the visiting sick infant. The providers were trained on how to fill up the form specifying the inclusion criteria of the study. The prescription forms were collected from the providers through the periodic visit and regular follow-up was done in an interval of three days. All data were range checked after collection. Double data entry was done in the Epi-info software. The data were analyzed using Stata version 11. Descriptive statistics – frequencies and percentages – were used for categorical variables. Data were presented in the form of tables, figures and pie charts. For quality assurance, piloting of the questionnaire was done in order to prevent the biases of the questionnaire.

From the IMNCI guideline, 24 symptoms were listed in the prescription pad and the only one injury was listed, other than the IMNCI guideline. By generating code for every symptom as per IMNCI guideline, the diagnosis was divided into 11 categories including injury. Diagnosis of diarrhea was done from the symptoms restless, irritable (a), sunken eyes (b), skin pinch goes back slowly (c), lethargic or unconsciousness (d). The infants diagnosed some dehydration (any two from a, b, c), severe dehydration (any two from b, c, d), severe persistent diarrhea (diarrhea lasting 14 days or more) and dysentery (blood in stool).

Diagnosis of possible bacterial infection was carried out from the symptoms umbilicus red or draining pus (e), pus discharge from ear (f), less than 10 skin pustules (g), convulsions (h), fast breathing – 60 breaths per minute or more (i), severe chest in-drawing (j), nasal flaring (k), grunting (l), more than 10 skin pustules or a big boil (m), axillary temperature 37.5°C or above or feels hot to touch (n), temperature less than 35.5°C or feels cold to touch (o), lethargy or unconscious (p) and less than normal movement (q). The infants diagnosed local bacterial infection (any one of the symptoms from e, f, g) and possible serious bacterial infection (any one of the symptoms from h to q). Diagnosis of feeding practice was carried out such as not suckling breast effectively, less than eight breastfeed within 24 h, receiving other foods or drinks, and ulcer or white patches in the mouth. Diagnosis of jaundice, fever, injury and other symptoms like vomiting, malaria, cough and cold was done. Symptomatic diagnosis as per IMNCI symptoms for possible bacterial infection, diarrhea and feeding problem were done; treatment patterns such as prescription of oral rehydration salt (ORS), zinc, vitamins, painkillers, paracetamol and antibiotics; and referral services for sick infants were collected from 15 untrained providers.

Ethical clearance was taken from the institutional ethical committee of Regional Medical Research Centre, Bhubaneswar. Permission was taken from the concerned local authority. Consent was taken from the study participants and it was informed to them that this study will not anyway harm them rather it will benefit the community; data will be used for result interpretation and sharing.

## Findings

A total of 15 providers participated in this study. The mean age of the participants was 38 (range 23–50) years. Out of 15 participants, 9 were graduate – 3 science, 5 arts and 1 commerce; 3 had an intermediate degree in science; and 3 of them had a high school education. Among all of them, only three had a professional qualification in Regular Medical Practitioner (RMP). Eleven of them have no other occupation than health-care practices, whereas two of them are involved in teaching and two involved in paramedical services. Only two participants had more than two years of professional experience and the remaining 13 had more than 10 years of experiences.

The mean distance from the provider’s clinic from sub-center is 4 km with a range of 200 m to 15 km. The mean distance between primary health-care center and the provider’s home is 10 km with a range of 0.5–35 km. The distance to community health center from the provider’s clinic was 13 km (range 0.5–26 km). Out of 15 participants, 9 providers told that there is no private hospital within 10–15 km radius of the location of their clinic. The mean average distance from patient’s location to the untrained provider’s is 13 km (range 5–30 km).

The average number of patients treated per day by one provider is 12 (range 2–45), whereas the average number of patients treated per week is 68 with range 6–275. The average number of infants treated per day is 2 (range 0–9), whereas the average number of infants treated per week is 13 (range 2–70). Out of 15 providers, 12 are providing 24-h services and all have medicines available at stock.

Around 6% of infants had consulted public hospitals before consulting the concerned providers. About 76% of infants had preferred to concern untrained providers directly at first step and 18% of infants were referred from other untrained providers. The results showed that about 94% of the parents in rural areas prefer untrained providers as the first choice of care in case of sick infants.

**Figure 1.**
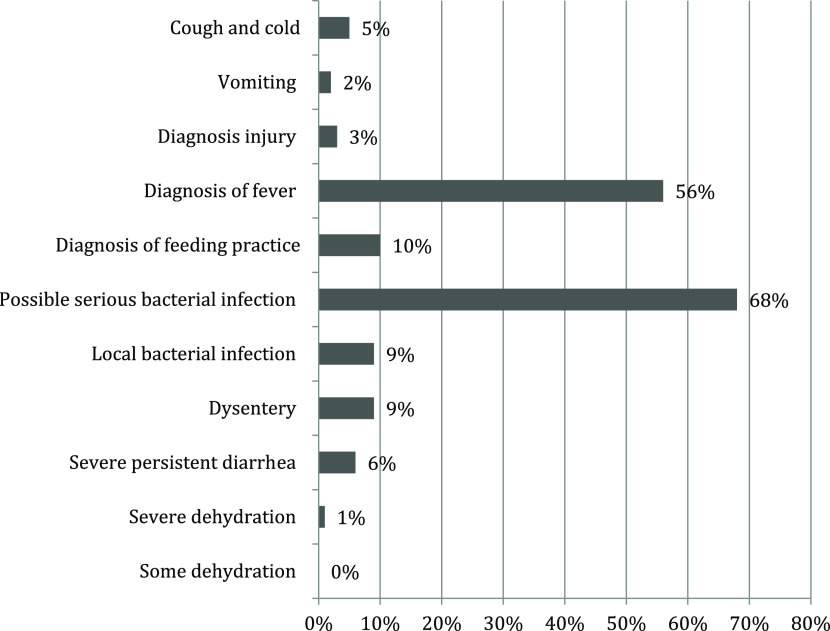
Detail diagnosis patterns of untrained providers in case of sick infants

Out of 337 sick infants, 59 infants (18%) were measured for temperature, 5 infants were measured for weight and none of the infant was measured for height. About 68% of the infants were diagnosed with a possible serious bacterial infection, 9% of infants were diagnosed with local bacterial infection and 56% of infants were diagnosed with fever. About 9% of the infants were diagnosed with dysentery – ‘blood in stool’, 6% severe persistent diarrhea and only 1% severe dehydration. However, none of the infants diagnosed with some dehydration. Around 10% of infants were found with feeding problems including not suckling breast effectively, less than eight times breastfeeds during 24 h, receiving outside food and ulcer or white patches in the mouth. Only 2% were diagnosed with vomiting, 5% suffered a cough and cold, and 3% had an injury. In Figure 1 detailed diagnosis patterns are given.

In Table 1 the treatment patterns of untrained providers for sick infants are presented. It was found that among the severe persistent diarrhea-diagnosed infants, 76% were prescribed ORS, 48% zinc and 62% antibiotics. Among the infants those who diagnosed with dysentery – 74% prescribed ORS, 23% zinc and 71% antibiotics. Both in the case of severe persistent diarrhea and dysentery, 19% were treated only with analgesics; paracetamol was prescribed to 29%.

**Table 1.** Treatment patterns of untrained providers in case of sick infants

Diagnosis as per IMCI guideline	Treatment patterns					
	ORS *n* (%)	Zinc *n* (%)	Vitamin *n* (%)	Paracetamol *n* (%)	Analgesics *n* (%)	Antibiotics *n* (%)
**Diarrhea**						
Severe dehydration (*n* = 1)	0	0	0	0	1 (100)	0
Severe persistent diarrhea (*n* = 21)	16 (76)	10 (48)	1 (5)	6 (29)	4 (19)	13 (62)
Dysentery (*n* = 31)	23 (74)	7 (23)	2 (6)	9 (29)	6 (19)	22 (71)
**Possible bacterial infection**						
Local bacterial infection (*n* = 30)	0	1 (3)	1 (3)	3 (10)	14 (47)	18 (60)
Possible serious bacterial infection (*n* = 228)	36 (16)	19 (8)	42 (18)	149 (65)	84 (37)	155 (68)
**Fever (*n* = 189)**	35 (19)	17 (9)	37 (20)	148 (78)	76 (40)	135 (71)
**Injury (*n* = 10)**	0	0	1 (10)	2 (20)	8 (80)	9 (90)
**Vomiting (*n* = 6)**	5 (83)	1 (17)	1 (17)	1 (17)	0	2(33)
**Malaria (*n* = 15)**	0	0	0	6 (40)	5 (33)	2 (13)
**Cough and cold (*n* = 17)**	4 (24)	0	3 (18)	7 (41)	7 (41)	15 (88)

Among all the sick infants, 30 infants had diagnosed with symptoms of local bacterial infections and 228 had diagnosed with symptoms of possible serious bacterial infections. Among the infants diagnosed with local bacterial infection, 60% were prescribed antibiotics, 47% were treated with analgesics and 10% treated with only paracetamol. In the case of possible serious bacterial infections, 68% of infants were provided with different generations of antibiotics and 37% were prescribed analgesics. Out of 189 fever-diagnosed infants, 78% were prescribed paracetamol, 71% were treated with antibiotics and 40% received analgesics. A total of 15 malaria cases had been diagnosed – among them, 40% received paracetamol, 13% antibiotics and 33% analgesics. In a total of 17 diagnosed with cough and cold, 88% (15) infants were prescribed antibiotics, whereas both paracetamol and analgesics were provided for 41% (7). Among the infants diagnosed with cough, 24% received ORS and 18% were provided with vitamins.

**Figure 2.**
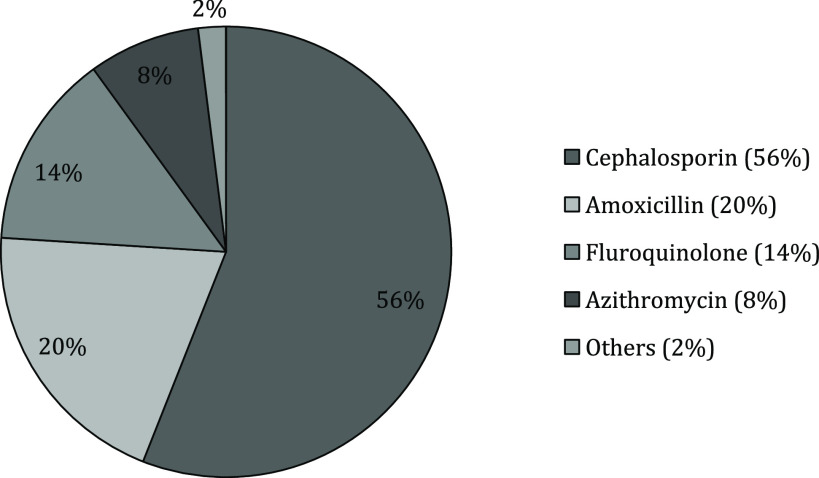
Antibiotics prescribed by untrained providers for the different symptoms of sick infants

Figure 2 shows the antibiotics prescribed by the providers for the different symptoms of sick infants. The commonly prescribed antibiotics were categorized according to the generic names of antibiotics – cephalosporin, amoxicillin, azithromycin and ofloxacin. A total of 61% (204) of sick infants were prescribed antibiotics; cephalosporin was prescribed for the highest percentage (56%) followed by amoxicillin (20%) and fluoroquinolone (ofloxacin and ciprofloxacin) 14%, and azithromycin.

Out of 337 sick infants, those who were treated by untrained providers, only 23% were referred to the trained providers either at public or private hospitals. Among the referred sick infants majority (55%) of them were referred to a district headquarter hospital or sub-divisional hospital, 36% community health centers, 5% primary health center and 4% were referred to private hospital.

## Discussion

This is the first study that showed the diagnosis, treatment and referral services provided by untrained providers for sick infants in rural settings. This study revealed that in rural Odisha most of the sick infants were seeking health care from the untrained providers. However, hardly any untrained providers were following the standard diagnosis and treatment guidelines; hence, improper diagnosis and irrational treatment were frequently observed. The antibiotic was the commonly prescribed medicine – 70% received antibiotics. The referral services were poor – 23% referred to trained health-care providers.

Several studies in India (Dror *et al*., 2011; Kanjilal, 2013; Gautham *et al*., 2014; Das and Mohpal, 2016) and in low- and middle-income countries showed that in rural settings a large volume of population were seeking health care from untrained providers (Mahmood *et al*., 2010; Sudhinaraset *et al*., 2013; Islam *et al*., 2014). Although, the community members preferred them because of convenience and affordability, most of them lacking knowledge on standard diagnosis protocol (Sudhinaraset *et al*., 2013).

The basic examination like measurement of temperature and weight of the sick infants is a foremost step of rationale treatment; weight measurement is decisive for dose calculation. This study found that temperature was not recorded in the case of 82% of infants and hardly any sick infant’s weight was measured. On the other hand, a study in Haryana, India, showed that most of the untrained providers had a stethoscope (100%) and clinical thermometer (99%); however, only 9% had weighing machine (Jarhyan *et al*., 2012).

A study in West Bengal, India, revealed that the most common diseases treated by untrained providers were diarrhea and gastro-enteric disorders (97%); they generally put saline and prescribed anti-diarrheal medicine (Kanjilal, 2013), which is similar to our findings. A study among informal health-care providers in Ujjain, India, found that for diarrhea only 58% prescribed ORS and 22% prescribed ORS with zinc; meanwhile, about 80% prescription contained other medicines like antibiotics, probiotics, racecadotril and antiemetics (Pathak *et al*., 2011). Almost all (99%) untrained providers in Haryana, India, prescribed and dispensed medicines, and all of them reported administering intravenous fluids for diarrhea; however, 15% used intravenous fluids to treat fever (Jarhyan *et al*., 2012). Similarly, a study in Nigeria reported that the prescription for sick infants by untrained providers depended on the severity of illness and the amount of money available for treatment (Rosamund *et al*., 2012). A study in North and South India revealed that inappropriate medicines and antibiotics prescription were higher among untrained providers (Gautham *et al*., 2014).

Our study found that above 70% of the sick infants received antibiotics in case of any type of diseases. A study in West Bengal found that the untrained providers were more likely to prescribe antibiotics; conversely, the dose and duration of antibiotics are not as per the standard protocols (Datta, 2013). Another study in Nigeria showed that 16% of patent medicine vendors prescribed antibiotics for fever (Rosamund *et al*., 2012). A study in Ujjain, India, reported that the untrained providers advised ORS with zinc significantly less often than the trained providers (Pathak *et al*., 2011). They also found that cephalosporin (57%) was commonly prescribed antibiotics, 71% prescribed one or more antibiotics and the odds of prescribing antibiotics were significantly higher in the presence of fever (Pathak *et al*., 2011), which is similar to our findings. Similarly, a study conducted in North and South India revealed that 30% of the patients received two or more antibiotics (Gautham *et al*., 2014). Most of the cases of the irrational prescription were provided by untrained providers as they do not have any formal training or any regulatory mechanisms to control their practice (Sudhinaraset *et al*., 2013). Hence, the ethical and legal aspects of rational treatment and regulation of unauthorized practice are one of the major health system fences for primary health care among rural sick infants.

This study came with the result that the untrained providers had referred 23% of sick infants, among them, 25% referred to other untrained providers, 5% to primary health centers and 70% to other higher facilities. Similarly, a study in West Bengal found that the untrained providers referred only 10% of sick infants to the formal health care providers (Kanjilal, 2013). A study in North and South India showed that the untrained providers refer patients to the primary health centers where adequate numbers of trained providers are available (Gautham *et al*., 2014).

In India, around two-third of the total population are living in rural areas. The problem of poor health-care access in rural areas is primarily due to unavailability of qualified doctors (De Costa & Diwan, 2007; George & Iyer 2013); rural areas have only 0.39 qualified doctors/1000 people against 1.33 for urban areas. Shortage of health providers has been identified as an important service delivery issue in rural settings. They constitute more than half of all the providers active in rural India (De Costa & Diwan, 2007; Sudhinaraset *et al*., 2013). The untrained providers conveyed that rural people have profound faith in them. The rural people were benefited in various ways. Meanwhile, the lacuna of primary health care in rural areas is being filled up by untrained providers (Datta, 2013). Therefore, it is challenging to ban untrained providers.

A systematic review on untrained providers in developing countries showed that greater access to formal providers and increased government oversight and regulation are the key steps to reduce irrational practice among untrained providers, and educational interventions, including capacity building exercises and training, were important for untrained providers (Sudhinaraset *et al*., 2013). A randomized control trial in rural India concluded that training of the informal providers enhanced the correct management of disease; however, it failed to reduce the unnecessary antibiotics prescription (Das *et al*., 2016). Hence, it is wise to control them for first aid for sick infants, to provide training on common disease algorithm and encourage them for referral services to a nearby health-care facility. A systematic review and meta-analysis revealed that integrated management of childhood illness (IMCI) training improves the skills of frontline health workers those who have basic qualification of matriculation (Nguyen *et al*., 2013; Sharma & Mukherjee, 2014). As per the Alma-Ata, the employment of lay health workers from the community should be trained to tackle specific tasks including provide first-level care, with appropriate referrals to secondary and tertiary health facilities. Hence, there is a necessity for training and restriction of treatment provided by untrained providers for sick infants (Mankar *et al*., 2012). They should provide first aid treatment to sick infants and refer them to the *Janani Shishu Suraksha Karyakarma* (JSSK) designated public facilities (Gopalan *et al*., 2014), where sick infants entitled for free provision of treatment, drugs, diet, blood transfusion and to-and-fro transport (Govt. of India, 2011; Govt. of India, 2012).

The limitation of this study was the identification of the untrained providers in the community and their willingness to participate in this study. There was not any systematic register or untrained providers association to enlist them. Hence, we identified them through interaction with community members and community mobilizers of the public health system such as accredited social health activists (ASHAs) and *Anganwadi* workers. Some of them were unwilling to participate in this study as they suspect that the government may take action against them for their practice. Furthermore, there was a lack of information regarding the number of infants seeking care from them. These factors affect the proper sample size and sampling. However, this study suggests the social mapping of untrained providers is noteworthy for future interventions on rational treatment for infants.

## Conclusion

In rural settings most of the sick infants were seeking health care from untrained providers. However, hardly any untrained providers followed the standard treatment protocol. Hence, this study suggests there is a need for training on common disease algorithm and treatment using standard guideline for untrained providers to reduce inopportuneness in the treatment of sick infants, which will promote early diagnosis and referral services to public health systems.
